# Linear and nonlinear interrelations show fundamentally distinct network structure in preictal intracranial EEG of epilepsy patients

**DOI:** 10.1002/hbm.24816

**Published:** 2019-10-18

**Authors:** Michael Müller, Matteo Caporro, Heidemarie Gast, Claudio Pollo, Roland Wiest, Kaspar Schindler, Christian Rummel

**Affiliations:** ^1^ Support Center for Advanced Neuroimaging (SCAN) University Institute for Diagnostic and Interventional Neuroradiology, Inselspital Bern Switzerland; ^2^ Department of Neurology, Inselspital, Bern University Hospital University Bern Bern Switzerland; ^3^ Department of Neurosurgery, Inselspital, Bern University Hospital University Bern Bern Switzerland

**Keywords:** epilepsy, epilepsy surgery, linear, network structure, nonlinear, quantitative EEG

## Abstract

Resection of the seizure generating tissue can be highly beneficial in patients with drug‐resistant epilepsy. However, only about half of all patients undergoing surgery get permanently and completely seizure free. Investigating the dependences between intracranial EEG signals adds a multivariate perspective largely unavailable to visual EEG analysis, which is the current clinical practice. We examined linear and nonlinear interrelations between intracranial EEG signals regarding their spatial distribution and network characteristics. The analyzed signals were recorded immediately before clinical seizure onset in epilepsy patients who received a standardized electrode implantation targeting the mesiotemporal structures. The linear interrelation networks were predominantly locally connected and highly reproducible between patients. In contrast, the nonlinear networks had a clearly centralized structure, which was specific for the individual pathology. The nonlinear interrelations were overrepresented in the focal hemisphere and in patients with no or only rare seizures after surgery specifically in the resected tissue. Connections to the outside were predominantly nonlinear. In all patients without worthwhile improvement after resective treatment, tissue producing strong nonlinear interrelations was left untouched by surgery. Our findings indicate that linear and nonlinear interrelations play fundamentally different roles in preictal intracranial EEG. Moreover, they suggest nonlinear signal interrelations to be a marker of epileptogenic tissue and not a characteristic of the mesiotemporal structures. Our results corroborate the network‐based nature of epilepsy and suggest the application of network analysis to support the planning of resective epilepsy surgery.

## 1 INTRODUCTION

For patients with focal drug‐resistant epilepsy surgical removal of brain tissue is a well‐established treatment option. To render patients seizure free and minimize adverse effects, localization of brain tissue necessary and sufficient to generate seizures (the epileptogenic zone EZ; Rosenow & Lüders, 2001; Lüders, Najm, Nair, Widdess‐Walsh, & Bingman, 2006) needs to be as accurate as possible. Since the EZ is a theoretical concept, it cannot in general be known if any (or even multiple) such zones exist. Only in patients who become seizure free postsurgically, the EZ (or at least one out of several) was contained completely in the resected tissue. Only about half of all patients who undergo resective therapy get completely seizure free (Engel class I) while the other half of patients continue to have seizures (approximately evenly distributed to the Engel classes II with considerably improved seizure control to IV without improvements) (de Tisi et al., 2011). To improve on this outcome, more accurate localization techniques for the seizure generating tissue are desired. Furthermore, novel forms of tissue removal as thermocoagulation (Cossu et al., 2017), laser ablation (Drane, 2018) and high‐intensity focused ultrasound (Quadri et al., 2018) could benefit even more from increased localization accuracy.

If, in the presurgical process of determining, the tissue to resect the available information of the noninvasive procedures (phase I) is not conclusive or the localized region is contiguous to eloquent cortex (e.g., the speech area), intracranial EEG (iEEG, phase II) is the gold standard to obtain information with superior spatial resolution and signal‐to‐noise ratio. Despite being labor‐intensive and prone to interrater variability, visual analysis by trained neurophysiologists/epileptologists is still the method predominantly used in clinical routine to analyze these signals. The signals showing the first continuous epileptiform activity constitute the seizure onset zone (SOZ), which is then used as a proxy for the EZ. However, the considerable portion of suboptimal outcomes suggests that classic approaches treating signals mostly as if they were isolated from each other could be too simplistic to capture the essential information in all cases. Instead, novel quantitative approaches incorporating interrelations among different parts of the epileptic brain might be necessary. Besides, automated analyses could abbreviate the labor‐intensive process of visual analysis and introduce more objectivity into it.

In the past, there has been research on numerous quantitative approaches to analyze iEEG signals to assess their potential to indicate epileptogenicity of the underlying tissue and thus be able to predict resection targets. A signal characteristic considered over the last two decades as a potential marker of the EZ were high frequency oscillations (HFOs) but meta‐analyses could not confirm an indicative role in epilepsy surgery decision‐making (Gloss, Nolan, & Staba, 2014; Höller et al., 2015). Moreover, two very recent studies, one retrospective (Roehri et al., 2018), and one multicenter prospective (Jacobs et al., 2018), both came to the conclusion that HFOs are not reliable markers in predicting the epileptogenic zone. These findings suggest that HFOs alone will not be sufficient for this task and other markers need to be considered.

There is growing evidence of a network‐based nature of epilepsy, meaning the interplay of several regions rather than a single focus is responsible for seizure initiation and propagation (Engel et al., 2013; Kramer & Cash, 2012; Scott et al., 2018; Shih, 2019; van Diessen, Diederen, Braun, Jansen, & Stam, 2013). This motivated efforts to move toward getting a multivariate view by capturing and analyzing dependences between signals and their changes. One can then interpret epochs of recordings as time‐varying networks where EEG channels constitute nodes and relations are defined by some bivariate measure (Rings, von Wrede, & Lehnertz, 2019). Subsequently, graph theory can be applied to identify critical nodes and subnetworks. Several studies have shown relations between such salient nodes and the resected brain tissue and its related postsurgical outcome (see, e.g., Jung et al., 2011; Wilke, Worrell, & He, 2011; van Mierlo et al., 2013; Zubler et al., 2015).

Network‐based approaches always involve the selection of an interrelation measure to define the strength (and in some cases also the direction) of the interaction between any pair of nodes. Different measures capture different signal characteristics and thus associated results must always be considered specific to the chosen dependence measure (and obviously also to further parameter choices). Incorporating multiple complementary types of interrelations in parallel might help to get a more complete view of the ongoing interactions and thus also contribute to improve on the outcomes in epilepsy surgery.

Recently, Rummel et al. (2015) used four measures corresponding to four different main categories of signal analysis: two univariate measures (one linear and one nonlinear), surrogate‐corrected Pearson's cross‐correlation as a multivariate linear measure, and surrogate‐corrected mutual information as a multivariate nonlinear measure. They calculated the contribution of each iEEG channel during different periictal phases and selected salient channels in a data‐driven manner for each measure separately. For three measures, the two univariate and the nonlinear multivariate, the fraction of these channels in the resected brain tissue was significantly enlarged if the patient became seizure free after surgery. The nonlinear multivariate method also had a higher average fraction of exceedingly high values within the resected tissue than the linear multivariate method in all outcome classes.

Preceding work has demonstrated a clear difference in linear and nonlinear connections after surrogate correction (Andrzejak, Chicharro, Lehnertz, & Mormann, 2011; Andrzejak, Schindler, & Rummel, 2012; Rummel et al., 2011; Rummel et al., 2015). Here, we expand the analyses on the linear and nonlinear interrelation matrices of (Rummel et al., 2011, 2015) by contrasting them regarding their structure and the distribution of the interrelations, in order to test the hypothesis that nonlinear excess interrelations between iEEG signals are different compared to linear ones and provide additional important information to identify epileptogenic brain tissue.

To increase the homogeneity of our data, we selected a subgroup of patients who all received a standardized bilateral depth electrode implantation targeting the mesiotemporal structures. We point out the fundamentally different structure of the two types of networks (linear and nonlinear) using assortativity, a graph theory measure quantifying similarity of connected nodes. We then analyze both types of interrelations based on their range, that is, if they act predominantly within or between hemispheres. We complement the characterization of the linear and nonlinear interrelations by calculating their strengths in dependence on their location and on their relation to the resected brain tissue. The entirety of our findings suggests a conception of seizure related activity where linear and nonlinear interrelations possess fundamentally different roles.

## 2 METHODS

### 2.1 Patients and data

In this study, we included 20 consecutive patients with drug‐resistant epilepsy who received implantation of depth electrodes targeting the mesiotemporal structures during evaluation for epilepsy surgery at the Inselspital Bern (10 female, median age 37.5 years, IQR 20.5 years, range 13–69 years). More detailed patient data are given in Table 1. Fifteen patients already had a resection at our center with a follow‐up of at least 3 months. In those, the resected areas and the electrodes recording from these tissues were determined via corregistration of a presurgical MRI, a postimplantation CT, and a postsurgical MRI (a detailed description of this procedure can be found in the study by Rummel et al., 2015). Nine patients who underwent resective surgery became completely free of disabling seizures (Engel outcome class I), three patients have rare disabling seizures since surgery (Engel class II), while in three patients, there was no improvement following surgery (Engel class IV). We did not exclude patients with a rather short follow‐up time to have as many patients as possible despite our restrictive inclusion criterion regarding the implantation scheme.

**Table 1:** Patients included in this study

	Engel class	Follow Up (month)	Seizure onset	MRI	# of tot. Ch.	# of std. ch. AmR/HiR/AmL/HiL	# of Res. Ch.
Patient							
1	II	4	MT (L)	Hippocampal sclerosis	48	8/8/8/8	7
2	I	4	MT (R)	Hippocampal sclerosis	32	8/8/8/8	5
3	I	3	MT (R)	Hippocampal sclerosis	38	7/8/0/8	8
4	II	24	MT (L)	Nonlesional	32	8/8/8/8	9
5	I	9	MT (R)	Mesiotemporal sclerosis	32	8/8/8/8	7
6	I	41	MT (R)	Hippocampal sclerosis	38	10/10/10/8	9
7	I	14	MT (R)	Hippocampal sclerosis	32	8/8/8/8	4
8	I	12	MT (L)	Hippocampal atrophy	31	8/8/8/7	7
9	II	3	MT (L)	Postischemic cyst	29	7/6/8/8	2
10	I	3	F (L)	Nonlesional	76	8/8/8/8	0
11	I	4	MT (R)	Hippocampal atrophy	31	8/8/8/7	0
12	I	19	OF (L)	Posttraumatic lesion	88	8/8/8/8	0
13	IV	6	MT (R)	Nonlesional	76	12/8/0/8	3
14	IV	11	Bitemporal	Mesiotemporal sclerosis	32	8/8/8/8	14
15	IV	24	F (L)	Dysplasia	70	8/9/7/4	0
16			LT (L)	Dysplasia	64	0/8/8/8	
17			MT (L)	Thickened MT structures	32	8/8/8/8	
18			MT (R)	Hippocampal sclerosis	31	8/7/8/8	
19			MT (R)	Nonlesional	24	8/0/8/8	
20			Bitemporal	Hippocampal atrophy	32	8/8/8/8	

*Note*: Indicated is the outcome of the resective surgery according to the Engel classification scheme, the syndrome, laterality and etiology, the total number of contacts implanted (# of tot. ch.), the electrode‐wise number of channels recording from mesiotemporal structures (# of std. ch.) and the number of those channels associated with resected brain tissue (# of res. ch.).

Abbreviations: MT: mesiotemporal, F: frontal, OF: Orbitofrontal, LT: lateral temporal, R: right, L: left, Am*: electrode recording from the amygdala, Hi*: electrode recording from the hippocampus.

This study was approved by the Ethics Committee of the Kanton of Bern (project no. 2017‐00697). All decisions regarding the actual treatment of the patients (especially implantation and resection) were made solely on clinical grounds prior to this study and all patients gave written and informed consent that EEG and imaging data may be used for research purposes.

Of each patient, we analyzed recordings containing the first two artifact‐free seizures occurring during iEEG monitoring and in order to maximize homogeneity we concentrated our analysis on those signals recorded from the depth electrodes implanted into the mesial temporal lobes even though some patients had individualized additional electrodes implanted. In addition, permanently artifact corrupted channels were excluded (<5% of channels). Decisions on artifacts and seizure onset and termination were made by experienced epileptologists/electroencephalographers (K.S. and H.G.) based on visual inspection.

Since the iterative generation of surrogate time series (see below) is computationally expensive, continuous analysis of interictal EEG with the chosen methods is prohibitive. Rather we needed to restrict our analysis to segments of few minutes duration. Kuhnert, Elger, and Lehnertz (2010) have found that functional networks calculated from interictal EEG exhibited large circadian variations, whereas epileptic seizures only caused smaller variability. To avoid bias when selecting the iEEG epochs, we selected 3 min immediately preceding the clinical seizure onset. This segment is uniquely defined relative to the seizures and serves as a very relevant baseline for visual EEG analysis by the clinical experts. In contrast to ictal data, the immediate preictal segment avoids artifacts that might be caused by seizure manifestation.

In summary, we included two recordings of 20 patients yielding 40 preictal epochs in total. For 24 epochs, we know the channels recording from tissue that was later resected causing a favorable outcome (Engel Classes I and II, Patients 1–12) and, for 18 epochs, these channels were located in the mesiotemporal structures (Patients 1–9).

### 2.2 Regions of interest

To contrast different regions regarding the strength of connectivity, we separated interrelations in various ways.

In all patients, we separated interrelations according to if they occurred between channels inside the hemisphere of seizure onset (focal hemisphere), between channels inside the contralateral hemisphere (nonfocal hemisphere), or between channels of different hemispheres. Moreover, in the subgroup of patients who underwent resective surgery targeting the mesial temporal areas with favorable seizure control (Engel Classes I and II, Patients 1–9), we separated interrelations according to if they occurred between channels recording from the subsequently resected brain tissue, between channels recording from the brain tissue ipsilateral to surgery but left untouched by surgery, or between these channels recording from opposite regions.

Likewise, we grouped the node strengths in all patients according to whether the channels were recording from the hemisphere ipsilateral or contralateral to seizure onset and in Patients 1–9 also according to if they were recording from the subsequently resected brain tissue or from the untouched brain tissue ipsilateral to surgery.

### 2.3 Network construction

In this study, we analyzed undirected surrogate‐corrected linear and nonlinear interrelation matrices, calculated in a sliding windowed procedure from iEEG recordings as described in detail in the study by Rummel et al. (2015). In brief, the linear interrelations were determined by Pearson's zero‐lag cross‐correlation matrix. Nonsignificant elements were removed by statistical comparison with correlation matrices created from univariate iterated amplitude adjusted Fourier transform (IAAFT) surrogate time series (Schreiber & Schmitz, 2000) and significant ones were scaled to the range [−1,1]. Thus, elements of our linear interaction matrices had significantly larger absolute values than those of uncorrelated time series with identical amplitude distributions and power spectra.

The nonlinear interrelation matrices were determined by mutual information, a measure that quantifies the amount of information one variable provides about the other. Since mutual information is sensitive to both linear and nonlinear dependences alike, we used multivariate IAAFT surrogate time series with conserved Pearson correlation matrix (Schreiber & Schmitz, 2000) to account for linear interrelation effects. Elements of our nonlinear excess interaction matrices had significantly stronger mutual information than surrogate time series with conserved Pearson correlation. Hence, the matrices describe the nonlinear excess interactions, that is, the interaction that is not measurable by linear interaction measures.

In addition, we used shift surrogates (Netoff & Schiff, 2002) to test explicitly, whether interrelation effects measurable by mutual information are caused by nonlinearities in the signals (e.g., nonlinear autocorrelation) or in the interaction. Opposite to multivariate IAAFT surrogates, shift surrogates preserve the linear and nonlinear autocorrelation of the signals but not the cross‐correlation between them.

### 2.4 Network analysis

To maximize the homogeneity of the electrode implantation schemes across patients, we focused our analysis on those channels that were part of the depth electrodes bilaterally implanted into the mesiotemporal structures.

To assess the predominance and reproducibility of interrelation patterns across epochs and patients we performed a principal component analysis (PCA) (Jolliffe, 2002) of the surrogate‐corrected linear and nonlinear interrelation matrices, matching the focal, and nonfocal hemispheres of the standardized mesiotemporal implantation scheme. That is, channels of the four electrodes of the standardized implantation were arranged in all patients in the same sequence, first the focal hemisphere, then the nonfocal one. For the three dominant components of these laterality‐matched matrices, we calculated the explained variance and also their collectivity and symmetry (Müller, Baier, Galka, Stephani, & Muhle, 2005). To assess the similarity of two interrelation matrices, we performed Mantel tests with *N* = 10,000 random permutations (Mantel, 1967). This test quantifies whether the observed (positive) correlation of matrix elements can be by chance or not.

**Figure 1:**
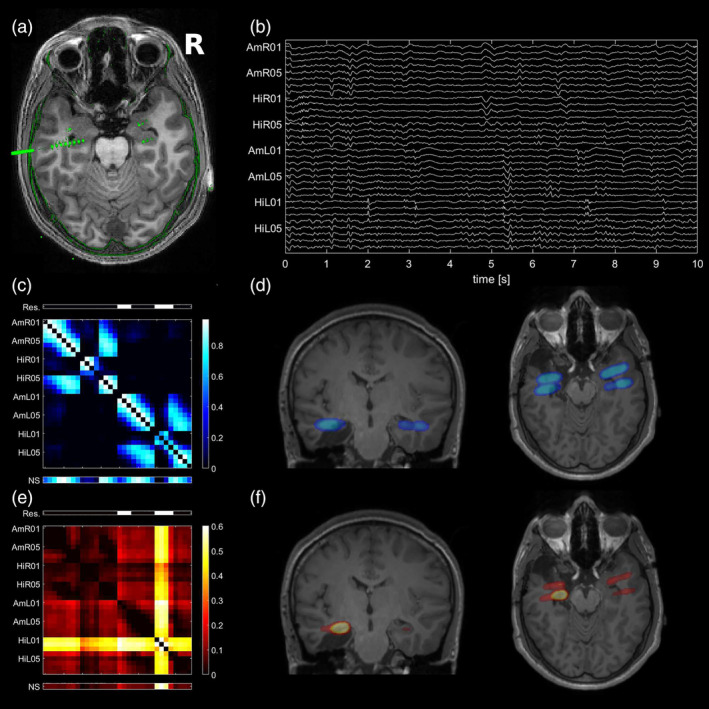
Typical example of the good outcome group (Patient 1): (a) preoperative T1‐weighted MRI coregistered to the postimplantation CT. The iEEG electrodes are visible as hyperintense artifacts in green. All eight contacts of the left hippocampal electrode are visible on the selected axial slice. In addition, contact AmL05 of the electrode recording from the left amygdala and contacts HR01 and AmR01 targeting the right mesiotemporal structures are visible. All MRIs are in neurological orientation, meaning that the right of the image corresponds to the right hemisphere. (b) Mesiotemporal iEEG recording of 10 s duration starting 3 min before seizure onset. Each channel is normalized separately to an identical range. (c) Average of all linear interrelation matrices of the analyzed preictal epoch. Blueish colors indicate linear interrelations throughout the manuscript. Above the matrix, the channels recording from brain tissue that was later resected are indicated by white bars (Res.). Below the matrix, the node strength is displayed (NS). (d) Transparent overlay of the node strengths of the linear interrelation matrix over the postoperative T1‐weighted MRI, coronal and axial slices. The extent of the resection is visible on the underlying MRI. To produce this image, 3D‐Gaussians with center at the electrode positions, a width of three voxels and integral proportional to the NS were summed up. (e) Average of all nonlinear excess interrelation matrices of the analyzed preictal epoch. Reddish colors indicate nonlinear excess interrelations throughout the manuscript. (f) NSs of the nonlinear excess interrelation matrix [Color figure can be viewed at http://wileyonlinelibrary.com]

To quantify the visually noticeable different structure of the linear and nonlinear networks (see Figure 1c,e), we calculated the networks' degree assortativity (Newman, 2002, 2003), a global (network wide), parameter‐free measure from graph theory not requiring any additional assumptions. Moreover, it is well defined for unconnected networks and since it is the Pearson correlation coefficient of the node degrees of connected pairs of vertices it has an absolute scale and does not require normalization to enable comparison regardless network size or density. It captures the tendency of a network's nodes to be connected to nodes with a similar degree (positive assortativity) or to nodes with a very different degree (negative assortativity/disassortativity). It is thus sensitive to the extent of difference in the topological importance of nodes. In addition, we identified core nodes in the networks. We automatically separated the channels into core (i.e., strongly connected) and peripheral (i.e., weakly connected) ones. This was done according to a procedure presented in the study by Rummel (2008) by sorting all channels by their node strengths and identifying the largest difference between two adjacent values on the linear and the logarithmic scale. We then calculated the fractions of the core nodes falling into the focal hemisphere and where applicable to the resected tissue.

Then, we analyzed the mean absolute linear or nonlinear interrelations according to their hemispheres (see Section 2.2). This analysis allowed the inclusion of all epochs from all patients, regardless of whether surgery was finally performed. In the subgroup of patients who underwent resective surgery targeting the mesial temporal areas with favorable seizure control (Engel Classes I and II, Patients 1–9), we additionally contrasted mean absolute interrelations separated with respect to the resected area (see Section 2.2).

Moreover, we quantified the total amount of interrelations a specific channel had using the node strength (i.e., the mean absolute matrix element connecting one node with all other nodes). We analyzed the node strengths groupwise with respect to the hemispheres and where applicable with respect to the resected area.

### 2.5 Statistics

We calculated the graph measures from surrogate‐corrected interrelation matrices derived from windows of 8 s duration displaced consecutively by 1 s over an epoch of 180 s directly preceding the visually determined seizure onset. These values were then averaged epoch‐wise for subsequent tests. For the PCA analysis and the identification of core nodes, we used the average of all matrices of an epoch. Accordingly, tests including all patients have a sample size of *N* = 40 and tests including only patients which had surgery in the mesiotemporal structures with a favorable outcome have a sample size of *N* = 18.

Due to exclusion of channels permanently corrupted by artifacts, the number of nodes in different patients varied. In addition, the absolute values of linear and nonlinear interrelations differ between patients and epochs. To enable comparisons across multiple epochs and patients, we normalized all node‐related measures to the relative amount an entity (node/region) contributed in an epoch. That is, the actual interrelation strength in each entity was divided by the value that would be expected if the total amount of interrelation strength was uniformly distributed. Hence, the resulting normalized values were factors indicating the entities' relative strengths with values <1 if an entity was underrepresented and >1 if it was overrepresented.

We used nonparametric testing throughout this study since test sizes were rather small and distributions potentially skewed. For all statistics, we used a significance level *α* = 0.01. In situations where the median of more than two groups was of interest, we first applied a Kruskal–Wallis test. If the null hypothesis that all groups stemmed from distributions with the same median was rejected, we applied pair‐wise post hoc Mann–Whitney *U*‐tests to determine which medians differed. Bonferroni correction was used in these cases to account for multiple testing. If the central position of only two groups needed to be compared, we used Mann–Whitney *U*‐tests directly. To determine whether the median of a group deviated from an expected value, we used sign tests of the difference. In all boxplots, the central line indicates the median of the distribution, the first (*q*1) and third quartile (*q*3) are indicated by the bottom resp. top edges of the box and the whiskers comprise all data points in the range *q*1–1.5 * (*q*3 − *q*1) to *q*3 + 1.5 * (*q*3 − *q*1). Values beyond this range are displayed as dots.

## 3 RESULTS

### 3.1 Similarity of different surrogate‐corrected interrelation matrices

Within the same epoch, the Pearson correlation matrix corrected with univariate IAAFT surrogates and the mutual information matrix corrected with shift surrogates were highly similar, see Figures S1 and S3. Epoch‐wise Mantel tests confirmed quantitatively that the observed similarity cannot be due to chance (maximal *p* < 10^−4^ in *N* = 40 tests). Accordingly, results from subsequent analyses based on the linear resp. the shift surrogate‐corrected nonlinear interrelation matrices were very similar. In contrast, both were dissimilar to the mutual information matrices corrected with multivariate IAAFT surrogates (see Figure S2, minimal *p* = .146 in *N* = 80 Mantel tests, indicating that the observed correlation of off‐diagonal matrix elements is very likely due to chance). Unlike the shift surrogate‐corrected nonlinear interrelations, the multivariate IAAFT surrogate‐corrected nonlinear interrelations provide additional information over the linear interrelations. Due to these findings, we restrict the presentation of our results to the interrelation matrices corrected by IAAFT surrogates in the sequel, which were also used in the study by Rummel et al. (2015). However, we did account for the results of the shift surrogate‐corrected nonlinear interrelation matrices in our multiple comparison protocols.

### 3.2 Group results

In Figure 1, we present example data of Patient 1 who only has rare seizures since resection of the amygdala, the hippocampus, and anterior parts of the temporal lobe on the left. Figure 1a shows the electrode‐contacts visible in a single axial slice mapped onto the presurgical MRI via coregistration with the postimplantation CT. As shown in Figure 1b are 10 s of the preictal EEG signals of all channels of the standardized mesiotemporal implantation. The time‐averaged matrix of linear and nonlinear excess interrelations is shown in Figure 1c resp. 1e with the resected channels indicated as a bar above. Similarly, each channel's node strength (NS) is displayed as a bar below and also mapped onto postsurgical MRIs (Figure 1d for the linear interrelations and Figure 1f for the nonlinear excess interrelations). While the linear interrelations are distributed evenly across all recorded areas, the nonlinear excess interrelations are clearly concentrated in the channels recording from the left hippocampus, which was surgically removed. The resection removed 25.2% of the nonlinear excess and 8.9% of the linear NS present immediately before onset of the first seizure. Under uniform distribution, 14.6% were expected for this resection size. Thus, the cumulative nonlinear excess NS was 1.7‐fold overrepresented in the resected brain tissue, whereas the linear NS was underrepresented by a factor 0.6. Restricting this analysis to the four resected channels recording from the left hippocampus, the nonlinear excess NS was overrepresented twofold (16.9% as compared to 8.3%), whereas the linear NS was even stronger underrepresented (3.4%).

The star structure visible in the exemplary nonlinear excess interrelation matrix (Figure 1e) was present in the corresponding matrix of the majority of analyzed epochs (see Figure S2). We automatically identified core nodes in all epoch‐wise averaged linear and nonlinear excess interrelation matrices. The numbers of core nodes and their proportions in the focal hemisphere are listed for all epochs in Table SI. For patients who had surgery in the mesiotemporal areas with a favorable outcome (Engel Classes I and II) the core nodes of the nonlinear excess matrices were in 17 of 18 epochs completely in the focal hemisphere and in the resected brain tissue. For class IV patients, the core nodes of the nonlinear excess networks were in three of four epochs completely in the focal hemisphere and in two of four epochs in the resected brain tissue. The latter was the case in Patient 14 which is discussed in detail below. In patients without surgery in the mesiotemporal areas, the nonlinear excess core nodes were in 14 of 18 epochs completely in the focal hemisphere. In the linear matrices, no core‐periphery structure exists (see Figure S1) and accordingly no actual core nodes could be identified. The associated proportions were thus at chance level.

**Figure 2:**
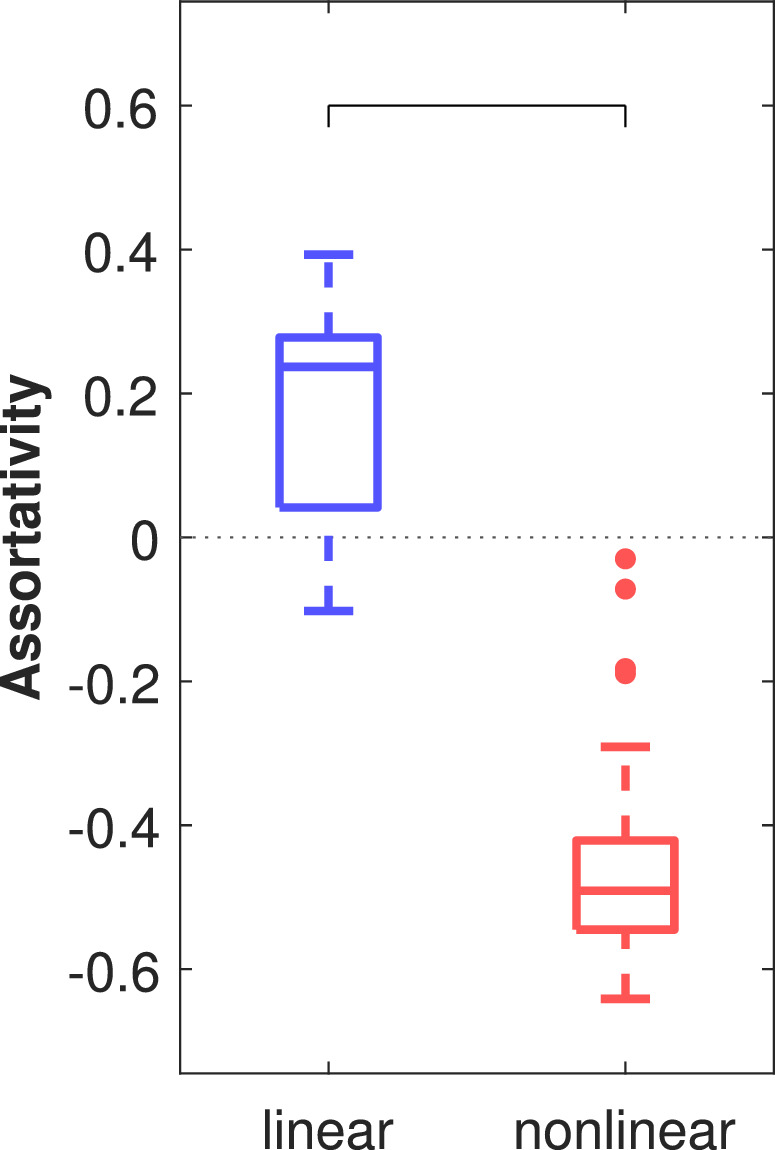
Average degree assortativities of linear and nonlinear excess networks constructed from preictal iEEG epochs (*N* = 40). The bracket indicates the significant difference in the medians of the two groups [Color figure can be viewed at http://wileyonlinelibrary.com]

The distribution of epoch‐wise averaged *degree assortativities* of the preictal linear and nonlinear excess networks are shown in Figure 2 for all 40 epochs to characterize their overall structure. The assortativity separates the linear and nonlinear excess networks and the hypothesis that linear and nonlinear excess networks have equal medians was rejected with very high significance (*p* < 10^−13^, Mann–Whitney *U* test). In all epochs, the nonlinear excess networks had on average a negative assortativity, while the linear networks typically had positive assortativity on average. The median was in both groups significantly different from zero (*p* < 10^−03^ for the linear and *p* < 10^−11^ for the nonlinear excess networks, sign test).

**Figure 3:**
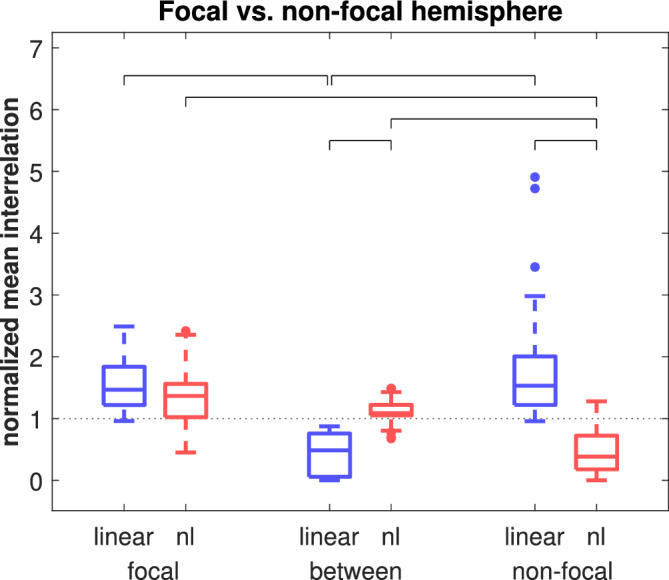
Linear and nonlinear excess interrelations within the focal hemisphere (focal), within the nonfocal hemisphere (nonfocal) and between the two hemispheres (between). Values were averaged and normalized epoch‐wise (see Section 2.5) (*N* = 40). Significant differences between associated group medians are indicated by brackets [Color figure can be viewed at http://wileyonlinelibrary.com]

To examine the interrelations depending on their spatial range, we distinguished them with respect to the hemispheres (see Section 2.2). For linear and nonlinear excess interrelations, the groupwise data are shown in Figure 3. The hypothesis of all groups having the same median was rejected (*p* < 10^−32^, Kruskal–Wallis test). In a post hoc analysis of pair‐wise tests we found (a) linear interrelations within both hemispheres were significantly stronger than between them (both *p* < 10^−13^, Mann–Whitney *U* test); (b) within the nonfocal hemisphere there were significantly stronger linear than nonlinear excess interrelations (*p* < 10^−11^), while there was no such difference within the focal hemisphere; (c) nonlinear excess interrelations were significantly stronger within the focal hemisphere than within the nonfocal hemisphere (*p* < 10^−10^); (d) nonlinear excess interrelations were significantly stronger between hemispheres than within the nonfocal hemisphere (*p* < 10^−12^); and (e) nonlinear excess interrelations between hemispheres were significantly stronger than the linear ones (*p* < 10^−12^). The number of multiple comparisons was 18: three types of interrelations (results from the shift surrogate‐corrected nonlinear interrelations are not shown) contrasted within three subdivided regions (nine tests) and each type of interrelation contrasted across the three regions (nine tests). The Bonferroni‐corrected significance level was thus 5.5 * 10^−4^.

**Figure 4:**
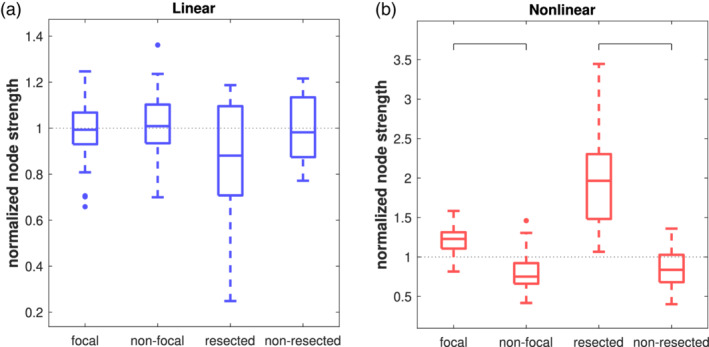
Normalized average node strength in linear (a) and nonlinear excess (b) networks, grouped by the hemisphere the channels were recording from (focal/nonfocal, *N* = 40) and those in the focal hemisphere by their association with the resected tissue (resected/nonresected, *N* = 18). For the latter only patients were included who had a resection in the mesiotemporal structures and had favorable postsurgical seizure control. For the nonlinear excess networks, region‐related grouped node strength was significantly different from each other (indicated by brackets) [Color figure can be viewed at http://wileyonlinelibrary.com]

We grouped the NSs (i.e., the total amount of linear resp. nonlinear excess interrelations present in any node) with respect to the hemisphere and where applicable with respect to the resected area (see Section 2.2). The results are shown in Figure 4. For the NSs of linear interrelations, there was no significant difference between the four groups (*p* = .177, Kruskal–Wallis test) and no deviation of any of the groups from the expectance under a uniform distribution of all linear interrelations (Figure 4a). There was no difference of linear interrelations between the hemispheres or between resected and nonresected areas. For the nonlinear excess interrelations (Figure 4b), the probability for NSs in all four groups coming from distributions with equal medians was *p* < 10^−14^ (Kruskal–Wallis test). The post hoc analysis found: the NSs of nonlinear excess interrelations were (a) significantly stronger in the focal than in the contralateral hemisphere (*p* < 10^−10^, Mann–Whitney *U* test) and (b) significantly stronger in the resected nodes than in the ipsilateral nonresected nodes (*p* < 10^−05^). Moreover, the NSs of nonlinear excess interrelations in the focal hemisphere and in the resected tissue were significantly larger than would be expected under a uniform distribution of all nonlinear excess interrelations (*p* < 10^−07^ resp. *p* < 10^−05^, sign test) whereas in the nonfocal hemisphere they were significantly weaker (*p* < 10^−07^). Within the resected brain tissue the median overrepresentation of nonlinear excess NS was 1.97 (range 1.07–3.45), whereas in the remaining ipsilateral channels, it was 0.84‐fold underrepresented (range 0.40–1.36). The number of multiple comparisons was 6: nonlinear excess interrelations contrasted according to two regional subdivisions (two tests) and all subdivisions contrasted against the expected value (four tests). The Bonferroni‐corrected significance level was thus 0.0017.

**Figure 5:**
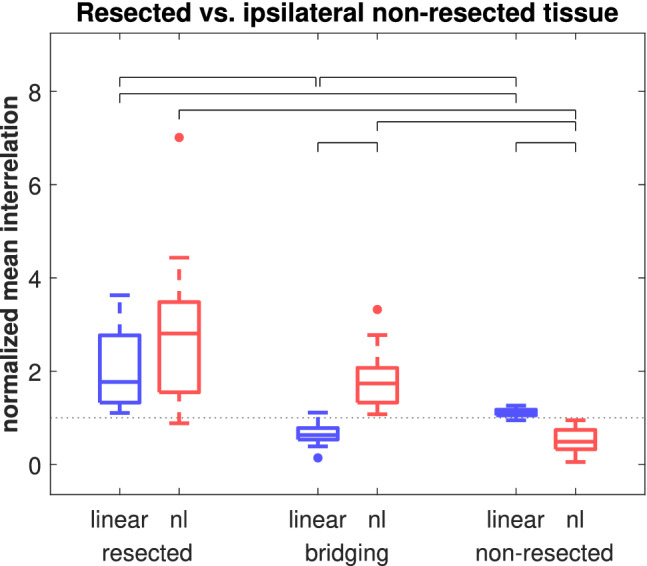
Linear and nonlinear excess interrelation within the resected tissue (resected), within the nonresected tissue of the ipsilateral hemisphere (nonresected) or linking those two areas (bridging). Only patients with a resection in the mesiotemporal structures and favorable outcome were included (*N* = 18). Values were averaged and normalized epoch‐wise (see Section 2.5). Significant differences between associated group medians are indicated by brackets [Color figure can be viewed at http://wileyonlinelibrary.com]

In the 11 patients who underwent resective surgery targeting the mesiotemporal areas the mean percentage of channels recording from the resected area was 0.22 (range: 0.07–0.44). Nine of these patients had favorable outcome (Engel Class I or II), whereas two had unfavorable outcome (Engel IV). We applied group statistics only to the favorable outcome group. Individual results of the Class IV patients are presented below. Linear and nonlinear excess interrelations separated with respect to the resected area (see Section 2.2) are shown in Figure 5. The hypothesis that the distributions of all groups have the same median was rejected (*p* < 10^−15^, Kruskal–Wallis test). The post hoc analysis found (a) significantly weaker linear interrelations bridging the resected and the nonresected area than acting inside or outside the resected area (*p* < 10^−06^ resp. *p* < 10^−05^, Mann–Whitney *U*‐tests); (b) significantly stronger linear interrelations inside the resected area than outside (*p* < 10^−05^); (c) significantly stronger nonlinear excess interrelations acting inside the resected area and bridging outwards than among channels outside (both *p* < 10^−06^); (d) significantly stronger nonlinear excess than linear bridging interrelations (*p* < 10^−06^); (e) significantly stronger linear than nonlinear excess outside interrelations (*p* < 10^−06^). Analogous to the analysis by hemisphere (see above) the number of multiple comparisons was 18 and the corresponding Bonferroni‐corrected significance level was 5.5× 10^−4^.

### 3.3 Reproducibility within and between patients

**Figure 6:**
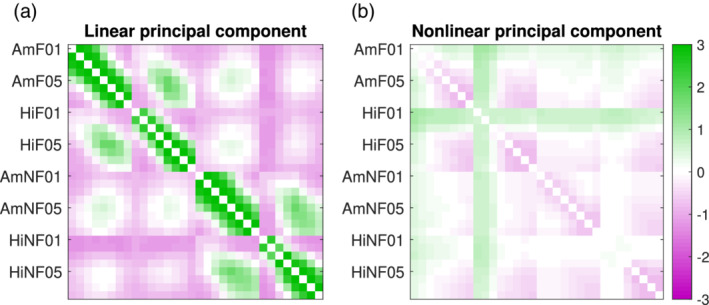
Principal component scores with highest explained variance of all epoch‐wise averaged and laterality‐matched IAAFT surrogate‐corrected linear (a) and nonlinear (b) matrices. The variance of the color‐coded component values corresponds to the explained variance of matrix elements. The bigger variance in the dominant component in (a) is due to the high reproducibility across patients. It shows high symmetry between hemispheres and predominantly short‐range connections whereas the dominant component of the multivariate IAAFT surrogate‐corrected nonlinear matrices (b) showed distinct differences between the hemispheres and strong long‐range connections [Color figure can be viewed at http://wileyonlinelibrary.com]

The epoch‐wise averaged linear interrelation matrices are displayed in Figure S1. Regardless laterality, large linear connections with high reproducibility between patients were mainly found within both hemispheres, whereas small and less reproducible connections were prevalent between them. A PCA of the laterality‐matched linear interrelation matrices revealed a dominant component explaining 63.7% of the total variance of matrix elements (Figure 6a). Collectivity was 0.95 and symmetry 1.0 (M. Müller et al., 2005), indicating that most individual linear interrelation matrices were combined constructively to obtain the dominant component. The dominant linear interrelation pattern showed that correlation decreased with distance between contacts on the same electrode as well as a weaker positive correlation between hippocampus and amygdala on the same hemisphere. The patterns were very similar between focal and nonfocal hemispheres. Interestingly, connections involving the first two contacts of the hippocampal electrodes on both hemispheres were weakly anticorrelated with the rest of the hippocampus and the amygdala. The same pattern was clearly visible in each one of the individual patients (Figure S1). The next two principal components explained only 6.3% and 5.6% of the total variance (collectivity: 0.42 resp. 0.37, symmetry: 0.13 resp. 0.02) indicating that a smaller fraction of epochs contributed, and contribution was not always constructive. Patterns were also symmetric between the focal and nonfocal hemispheres.

The epoch‐wise averaged nonlinear excess interrelation matrices are displayed in Figure S2. Long‐range interrelations were much more dominant and the dissimilarity between patients was much larger here than in the linear networks. In general, in some but not all patients, the first three contacts of the focal hippocampal electrode were prominently interacting with many other channels. These observations were well summarized by the dominant component of the PCA of the laterality‐matched matrices in Figure 6b (total explained variance 50.4%, collectivity: 0.23, symmetry: 1.0). Compared to Figure 6a, the symmetry between the focal and nonfocal hemispheres was absent. The dominant pattern consisted of long‐range nonlinear excess interrelations between the first two contacts recording from the hippocampus on the focal hemisphere and all other contacts. Similar but weaker interrelations were centered at the first contacts of the electrode recording from the amygdala on the focal hemisphere. The next two principal components of the nonlinear excess interrelation matrices explained 25.2% and 7.3% of the total variance (collectivity: 0.16 resp. 0.28, symmetry: 0.08 resp. 0.19). Components number 2 and 3 mainly consisted of nonlinear excess interrelations between the focal hippocampus and amygdala as well as long‐range connections of the focal hippocampus.

Without showing the results, we remark that we have repeated this analysis, overlaying the left and right hemispheres rather than the focal and nonfocal ones. The findings were similar and the differences between reproducible linear and specific nonlinear excess matrices were even more pronounced.

### 3.4 Patients with poor outcome: Individual results

Three of the included patients had Engel outcome Class IV after surgery. Because clinical characteristics in these patients were heterogeneous and patients with unfavorable outcome are most valuable for future improvement of computer‐assisted presurgical evaluation, we discuss the results separately instead of attempting group statistics. Where applicable and necessary, we repeated the analysis with the full channel set, including extra‐temporal locations.

Patient 13 is a 19‐year‐old female patient experiencing about one dyscognitive seizure a day despite prior surgery (selective amygdalohippocampectomy and parahippocampectomy on the right, 2 years earlier) and medication with several seizure‐suppressing drugs. Epilepsy duration was 7 years. The MRI showed an asymmetry of the posterior parts of the hippocampi (smaller on the right), a discrete malformation of the right amygdala as well as malformation of the right collateral sulcus and parahippocampal gyrus.

**Figure 7:**
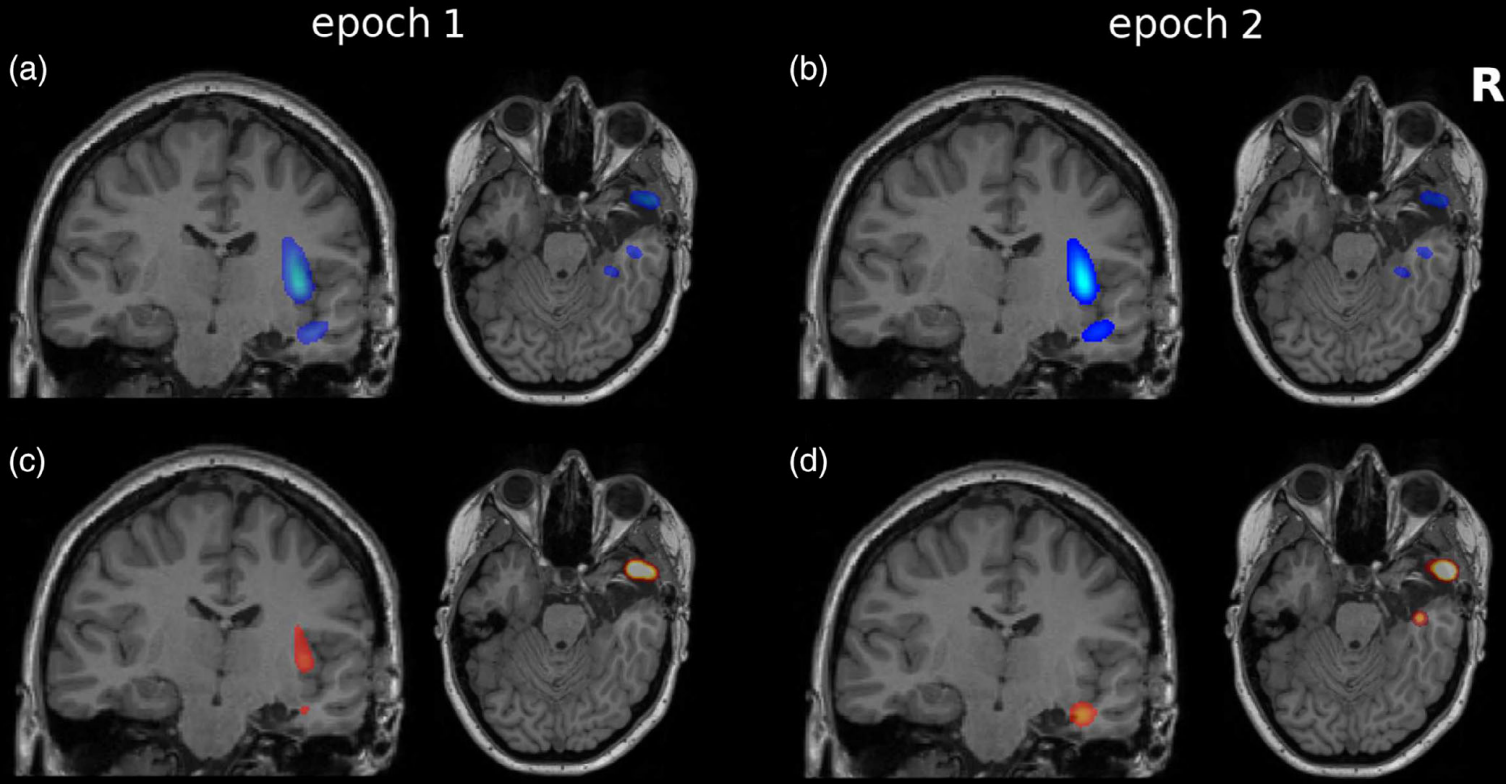
Transparent overlays of the node strengths of the linear (a,b) and nonlinear excess (c,d) interrelation matrices before seizure 1 (a,c) and seizure 2 (b,d) of Patient 13 over the postoperative T1‐weighted MRI, coronal, and axial slices. The extent of the resection is visible on the underlying MRI. For the corresponding matrices see Figure S4 [Color figure can be viewed at http://wileyonlinelibrary.com]

The semiology of seizures observed in phase I EEG monitoring was consistent with seizure onset in right mesiotemporal structures, which led to a direct first resection without phase II monitoring. Since the seizures did not reduce satisfactorily after surgery, eight depth electrodes were implanted into the hippocampus, amygdala, insula, temporo‐anterior and temporo‐polar regions on the right as well as into the left hippocampus (altogether 76 contacts, all free of permanent artifacts). During the four recorded seizures, a seizure onset in the remains of the right uncus and a stereotypical rapid propagation pattern to ipsilateral temporo‐anterior and temporo‐polar electrodes was observed. Propagation to the contralateral hippocampus was delayed and completely missing in seizure 3. Based on these findings, a second resection of the remains of the right amygdala, hippocampus, temporal pole and parahippocampal gyrus was performed (see Figure 7), but the seizure rate remained unchanged.

Quantitative analysis of preictal EEG yielded different findings before the first two seizures. Prior to seizure 1, nonlinear excess interrelations were observed mainly in the right temporal pole (see Figure 7c and Figure S4C) with sporadic interrelations also in the right amygdala and between the left and right hippocampus. The fraction of resected nonlinear excess NS was 31.2% and thus exceeded the expectation under uniform distribution (21.1%) by a factor of 1.5. Prior to seizure 2, nonlinear excess interrelations were predominant in the right hippocampus and temporal pole, see Figure 7d and Figure S4D. The resection comprised parts of the right amygdala and hippocampus and the right temporal pole, removing 55.3% of the nonlinear excess NS (overrepresentation by a factor 2.6). However, the tissue monitored by the right hippocampal contacts showing strong nonlinear excess interactions prior to seizure 2 were left intact, see Figure 7d and Figure S4D. Specifically, channel HiR01 cumulated 7.9% of the nonlinear excess NS as compared to an expectation of 1.3%.

Patient 14 is a 45 –year‐old female patient with dyscognitive seizures and epilepsy duration of 18 years. Prior to surgery, the patient perceived at least one seizure per month despite two seizure‐suppressing drugs. On the MRI discrete signs of mesiotemporal sclerosis on the right were identified. Conflicting with the MRI findings, the phase I EEG monitoring suggested seizure origin in the left mesiotemporal structures. To lateralize and localize the SOZ, a phase II EEG monitoring was performed with four depth electrodes targeting the bilateral mesiotemporal structures (32 contacts, all free of permanent artifacts). Altogether 14 seizures were recorded, 13 of which started in the right hippocampus according to visual EEG analysis. None of them propagated to the left hemisphere and neither were they noticed by the patient nor were clinical signs observable in the video monitoring which classifies them as “subclinical.” However, the SOZ of the 11th seizure was identified in the left hippocampus and amygdala. Semiologically, smacking and cramping of the right hand were observed. Interictal spikes were observed on both hemispheres. Because most of the seizure onsets were localized in the right hippocampus, which importantly also showed MR findings typical for sclerosis, a right‐sided selective amygdala‐hippocampectomy was performed “palliatively,” that is, expecting a significant reduction of seizure rate (Holmes, Miles, Dodrill, Ojemann, & Wilensky, 2003).

**Figure 8:**
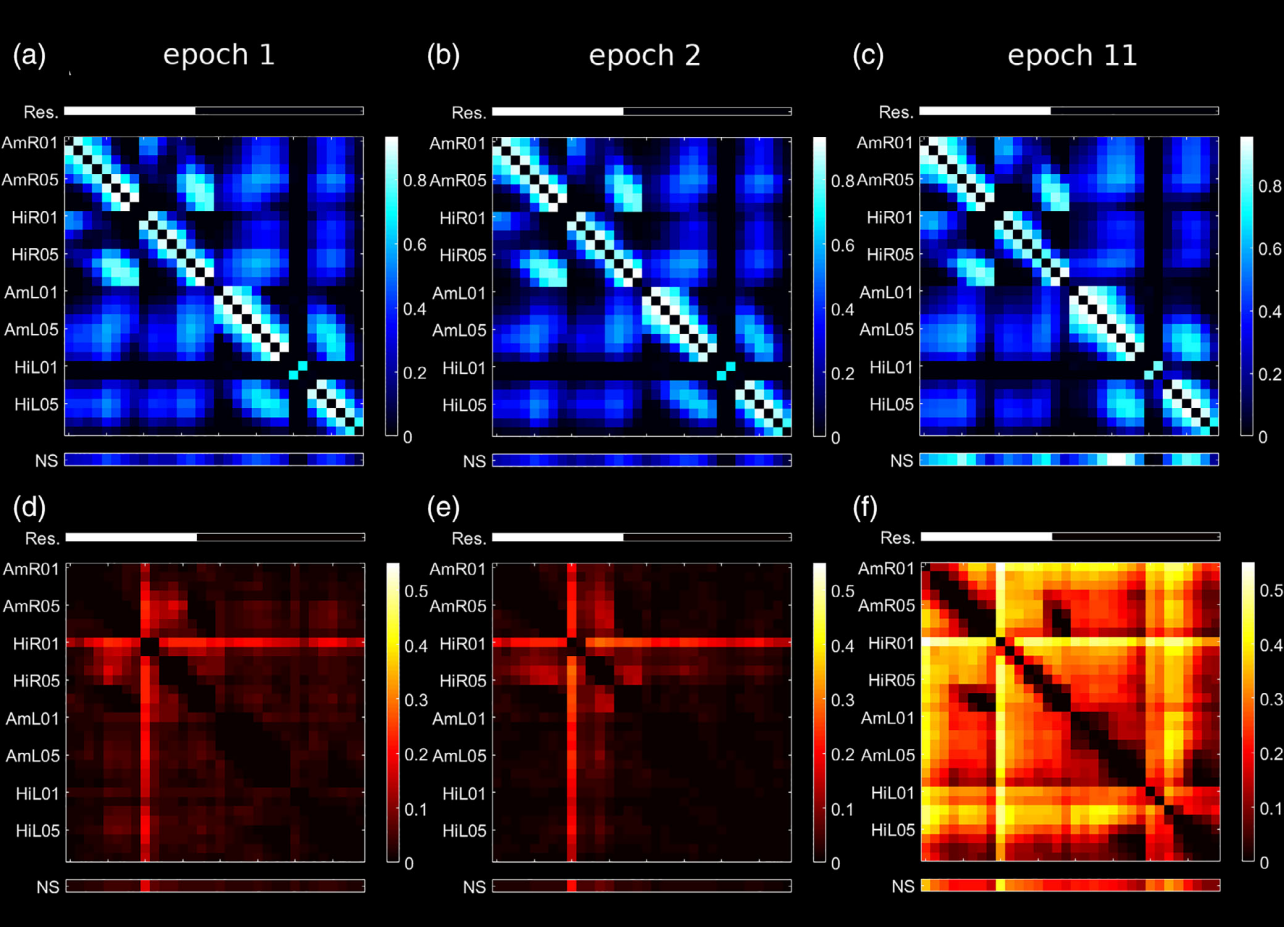
Average of all linear (a–c) and nonlinear excess (d–f) interrelation matrices of the analyzed preictal epochs 1 (a,d), 2 (b,e), and 11 (c,f) of Patient 14. Above the matrices, the channels recording from brain tissue that was later resected are indicated by white bars (Res.). Below the matrices, the node strength is displayed (NS) [Color figure can be viewed at http://wileyonlinelibrary.com]

Results of our quantitative analysis of preictal EEG prior to seizure 1 and 2 were very similar, see Figure 8a,b,e. Linear interrelations were equally strong in both hemispheres and connections between hemispheres were weaker than within them. The nonlinear excess interrelation NS was clearly peaked in channel HiR01 recording from the right hippocampus (which thus was the sole automatically identified core node). Surgery removed channels HiR01 to HiR06 recording from the right hippocampus as well as eight channels recording from the right amygdala (AmR01 to AmR08). Altogether this corresponded to 56.3% of the nonlinear excess NS as compared to 43.8% expected under uniform distribution. Channel HiR01 alone more than quadrupled the expected nonlinear excess interrelation strength (13.8% vs. 3.1*%*).

We also analyzed the seizure originating in the left mesiotemporal structures. Prior to seizure 11, the linear interrelation pattern was very similar to the situation found before seizures 1 and 2, see Figure 8c. However, the nonlinear excess interrelations differed from the earlier seizures. Rather than being peaked in a few channels recording from the right hippocampus, the NS was much more uniformly distributed and interrelations were similar within and between both hemispheres, see Figure 8f. While channel HiR01 still had the largest strength (5.8% as compared to an expectation of 3.1%), only 50.1% of the nonlinear excess NS present during this epoch were resected.

Patient 15 is a 31‐year‐old male patient experiencing focal seizures with and without secondary generalization. Epilepsy duration was 27 years. The MRI showed infratentorial atrophy, whereas potentially epileptogenic lesions were not detected. Before surgery, the patient experienced several seizures per day despite medication with several seizure‐suppressing drugs. The seizure semiology and phase I EEG monitoring with scalp electrodes suggested seizure origin in the left supplementary sensor‐motor area (SMA). To better localize the SOZ and to monitor seizure propagation pathways, an extended phase II EEG monitoring with additional electrodes outside the left SMA was performed. Altogether 10 depth electrodes were implanted symmetrically (96 contacts in total, 69 of them inside brain parenchyma and free of permanent artifacts). Four depth electrodes targeted the mesiotemporal structures of both hemispheres. Several clinical and subclinical seizures with stereotypical EEG pattern starting in the left SMA and rapid propagation to the contralateral hemisphere were recorded per hour. Seizure onset was localized to the left superior frontal gyrus. To reduce the seizure rate, surgery was performed in the left SMA, initially leading to almost seizure freedom. Six months after surgery, the seizure rate had increased again and 24 months after surgery was back to the presurgical state.

**Figure 9:**
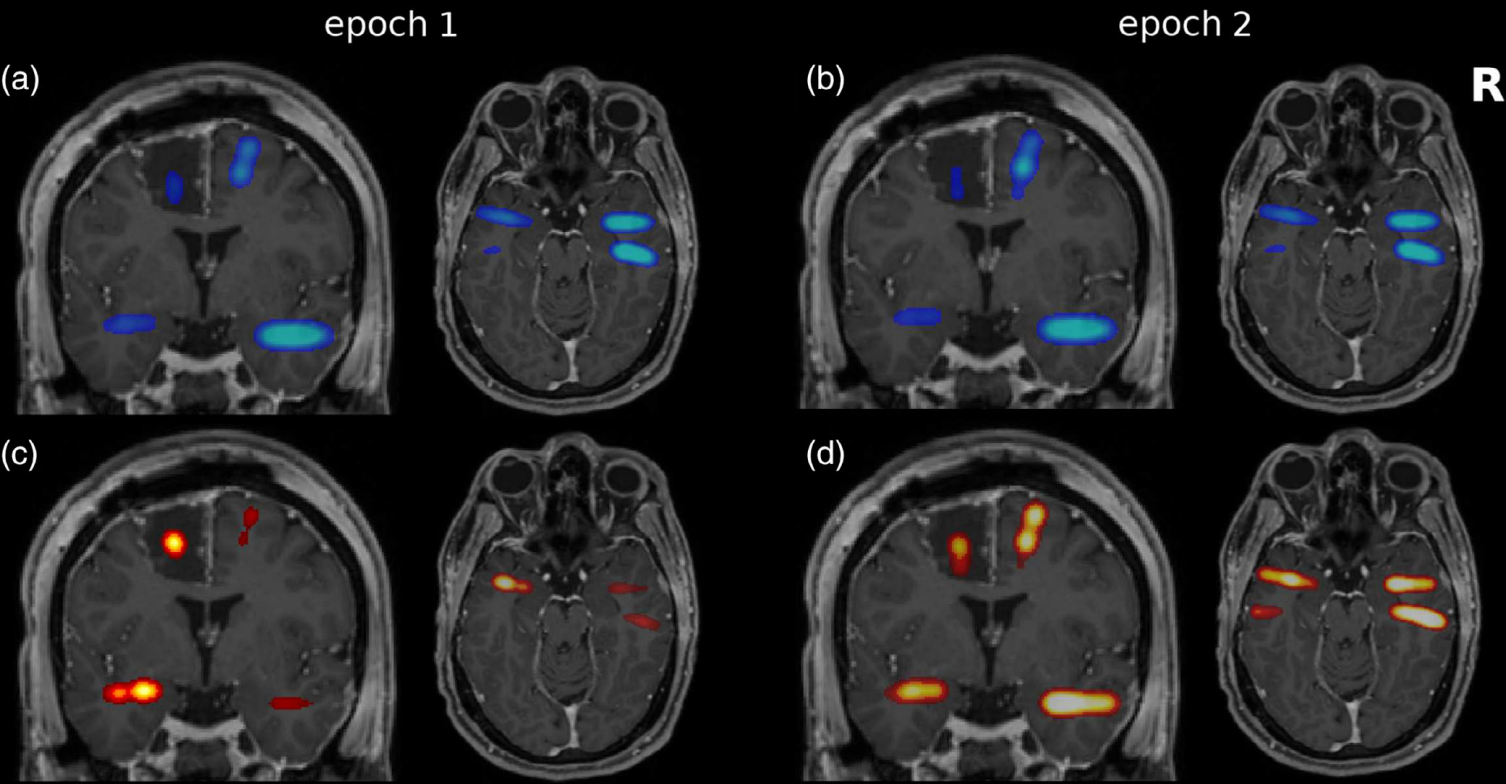
The same as in Figure 7 for Patient 15. For the corresponding matrices, see Figure S5 [Color figure can be viewed at http://wileyonlinelibrary.com]

Our quantitative analysis of preictal EEG revealed that linear signal interrelations were stronger and more extended in the right than in the left hemisphere, see Figure 9a,b and Figure S5A,B. Nonlinear excess interrelations in the standardized electrode implantation recording from the mesiotemporal structures were mainly found in the left amygdala (channels AmL01, AmL02, AmL04, AmL05, see Figure S5C), that is, ipsilateral to the visually determined seizure onset but not touched by the resection. Repeating our analysis with the full EEG data set revealed that the contacts in the left SMA indeed did interact nonlinearly (mainly channel SMAL05), see Figure 9c,d and Figure S5C. However, the percentage of nonlinear excess NS removed after surgery was only 13.3% and 8.7% immediately before onset of seizures 1 and 2. Before seizure 2, where the nonlinear excess NS was almost evenly distributed over all channels, this corresponded exactly to the expectation under uniform distribution, while it was only slightly overrepresented prior to the first seizure. In contrast, the cumulative nonlinear excess NS in the seven channels recording from the left amygdala tripled the expectation immediately before the first seizure (33.6% compared to 10.1%).

## 4 SUMMARY AND DISCUSSION

We examined surrogate‐corrected linear and nonlinear interrelations in the immediate preictal phase of patients with focal epilepsies to test the hypothesis that nonlinear excess interrelations between iEEG signals are different compared to linear ones and provide additional important information to identify epileptogenic brain tissue. The linear and nonlinear dependence measures were chosen based on preceding work, which demonstrated a clear difference in linear and nonlinear connections after surrogate correction (Andrzejak et al., 2011, 2012; Rummel et al., 2011; Rummel et al., 2015). Interrelations were calculated based on EEG signals obtained from bilateral depth electrode recordings and our main focus was on the mesiotemporal structures to enhance the homogeneity of our data set. We explored fundamental structural differences between linear and nonlinear excess networks and analyzed the interrelations according to their range and the regions that interact.

### 4.1 Main findings

The surrogate‐corrected Pearson correlation matrices and the mutual information matrices corrected with shift surrogates were highly similar visually (see Figures S1 and S3) as well as in Mantel tests. We conclude that the prominent difference between the linear and nonlinear interrelation matrices, corrected by univariate and multivariate IAAFT surrogates respectively, is *not* due to a different sensitivity of the linear and nonlinear interrelation measure to nonlinear autocorrelation of the signals. Rather, the correction by multivariate IAAFT surrogates is needed to become sensitive to the small but significant nonlinear excess interactions that are only measurable by mutual information (Rummel et al., 2011).

The interrelation patterns of our study were highly reproducible between different epochs of the same patient (Figures S1–S3. For the linear and the shift surrogate‐corrected nonlinear interrelations of the mesiotemporal depth electrodes, this was also true between different patients (Figure 6a). The nonlinear excess interrelation patterns (i.e., mutual information matrices corrected using multivariate IAAFT surrogates) on the other hand were patient specific (Figure 6b). Our observation for the linear interrelations is consistent with recent scalp EEG findings where a generic and very stable background pattern has been found (Müller, Rummel, Goodfellow, & Schindler, 2014 ; Olguín‐Rodríguez et al., 2018). We interpret this as an indication that the linear interrelations mainly reflect the mesiotemporal anatomy, which in our study was recorded in a standardized fashion. In contrast, nonlinear excess interrelations reflect the patient‐specific pathology and are not a property of the mesiotemporal structures. They are typically focused in a few channels with strong connections to most of the other recorded areas. In patients with a favorable postsurgical outcome, these core channels were mostly recording from subsequently resected tissue, whereas all patients without worthwhile improvement after surgery showed strong nonlinear excess interrelations in at least one epoch in tissue that was *not* resected. Our findings congruently suggest nonlinear excess signal interrelations to be a marker of epileptogenic tissue depicting pathologic activity and their removal to be strongly related to postsurgical improvement.

The graph measure *degree assortativity* specifies if network nodes prefer connections to nodes with a similar degree (positive assortativity for uniformly integrated nodes) or to nodes with a very different degree (negative assortativity for networks with pronounced differences in the topological importance of nodes). Our finding of consistently negative assortativity of the nonlinear excess networks (Figure 2) is induced by their star‐like structure having a few core nodes connected to most others while connections among noncentral nodes are only weak (examples shown in Figures 1e and 8d,e). A characteristic of such star‐structured networks is the low path length between any two nodes enabling fast transmission of information throughout them (Goñi et al., 2013). Star networks are particularly vulnerable to targeted attacks. Removal of the core nodes changes the network properties much more drastically than in more uniformly integrated networks (Albert, Jeong, & Barabási, 2000). The linear networks calculated at zero time‐lag were in contrast found to have a more block‐like structure where strong interrelations exist mainly locally between the channels on the same depth electrode and between the electrodes of the same hemisphere but rarely between the hemispheres. In agreement with the finding by (Bialonski & Lehnertz, 2013) this resulted in a positive assortativity in most cases (example shown in Figure 1c).

**Figure 10:**
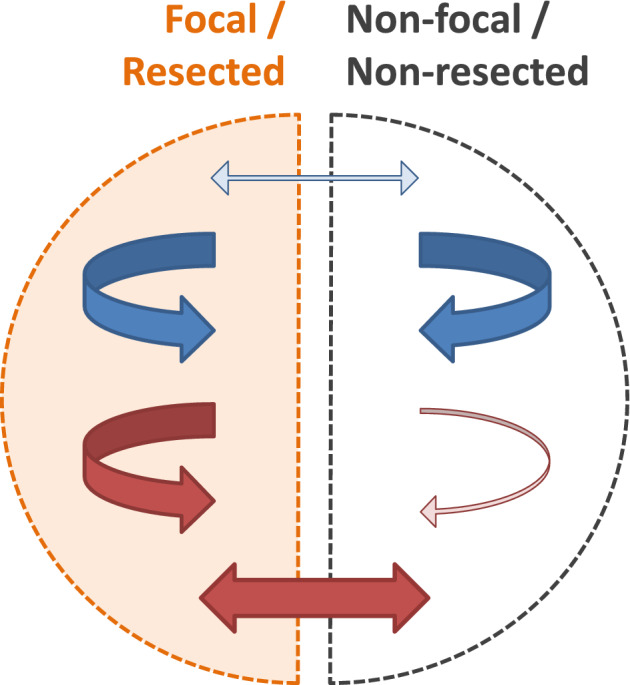
Schematic representation of our main results. Linear interrelations (blue arrows) are strongly (thick arrows) and equally present in the focal and nonfocal hemisphere. In surgically treated patients with a favorable outcome, the same is true in the resected tissue and the ipsilateral nonresected tissue. In both cases, linear interrelations between these regions are only weak (thin arrow). Nonlinear excess interrelations (red arrows) are significantly stronger in the focal hemisphere and in the resected tissue than in the nonfocal hemisphere resp. the ipsilateral nonresected tissue. Nonlinear excess interrelations between those regions are also strong [Color figure can be viewed at http://wileyonlinelibrary.com]

Our main results are summarized in Figure 10. Blue (red) arrows depict linear (nonlinear excess) interrelations and thin (thick) arrows depict weak (strong) mean absolute interrelations. First, when interpreting the two compartments as the focal and the contralateral nonfocal brain hemispheres, it sketches the results of Figure 3. Inside the focal hemisphere, both linear and nonlinear excess interrelations were strongly present. Inside the nonfocal hemisphere, mainly linear interrelations were present with nonlinear excess interrelations clearly diminished. Contrariwise, between both hemispheres, interrelations were predominantly nonlinear and linear equal‐time connections were clearly underrepresented. Apparently, linear equal‐time interrelations mostly constitute short‐range connections inside both temporal lobes while for the long‐range transmission between hemispheres nonlinear excess interrelations are mainly responsible.

Second, in the patients who have undergone successful epilepsy surgery, the compartments can also be interpreted as the resection area and the nonresected parts of the ipsilateral hemisphere. In this case, Figure 10 visualizes the results of Figure 5. Linear interrelations bridging the resected zone and the ipsilateral remainder were significantly diminished compared to the interrelations acting inside or outside but ipsilateral to the resected area. A similar result has been found earlier by Warren et al. (2010). In terms of linear interrelations, the focal zone appeared disconnected from the remaining brain areas covered by our standardized implantation scheme. The same analysis using the nonlinear excess interrelations showed again a different picture. Whereas there were strong nonlinear excess interrelations inside and bridging, they were clearly underrepresented among channels outside but ipsilateral to the resected area. Bridging connections were thus predominantly nonlinear (Figure 5).

Finally, regarding the channels' NS, we found no difference between the linear interrelations in both hemispheres and no difference between the resected and the nonresected tissue (Figure 4a). Hence, the total amount of linear interrelations was not related to the focal zone. In contrast, regarding nonlinear excess interrelations, we found significantly stronger nonlinear excess interrelations in the focal hemisphere compared to the nonfocal hemisphere and even stronger nonlinear excess interrelations in the channels recording from the resected area (Figure 4b). Thus, the excessive presence in the focal hemisphere was concentrated in these signals.

### 4.2 Patients with insufficient seizure control after surgery

In patients who became seizure free after surgery, the epileptogenic zone has been removed by definition. In consequence, an upper bound for the epileptogenic zone included in the resected brain tissue is known. In contrast, we can only speculate what would have happened if surgery had been performed differently in patients with unfavorable outcome. We had three such cases in our study, where the standardized mesiotemporal implantation scheme was an inclusion criterion. In none of them the predominantly local linear interrelation pattern differed from the one found in the good outcome group. The nonlinear excess interrelation patterns on the other hand allowed in all cases explaining the unfavorable outcome by strong nonlinear excess interrelations in at least one analyzed epoch, that were recorded from tissue left untouched by surgery.

In Patient 13, a channel accumulating a significant part of the nonlinear excess interrelation strength prior to the second seizure (HiR01) was left intact by surgery despite removal of a considerable fraction of the nonlinear excess interrelations and the right hippocampus. In the context of our findings in the good outcome group, we hypothesize that a slightly more aggressive surgery might have led to improved outcome in terms of postsurgical seizure control.

The observations made in Epochs 1 and 2 of Patient 14 were in full agreement with our findings in the good outcome group. A considerable part of the right mesiotemporal channels exhibiting nonlinear excess EEG interrelations prior to these seizures was removed. Based on the radiological suspicion of mesiotemporal sclerosis on the right and our findings in these two epochs alone, one could have expected a much better postsurgical outcome. However, before seizure 11, the only seizure with onset in the left mesiotemporal structures, nonlinear excess interrelations were widely distributed over all channels and almost equally strong on the left hemisphere (Figure 8f). We hypothesize that resection of the left mesiotemporal structures might have led to a reduction of the seizures the patient recognized and might thus have had greater improvement of the subjective perception of the patient. Since bilateral surgery was not an option, surgery on the right agreed with the MRI findings and the numerical proportion of recorded seizures.

In Patient 15, we observed that only a small fraction of the channels showing strong nonlinear excess interrelations was removed. Specifically, before onset of the first seizure, these were expressed much more strongly in the signals recording from the left amygdala than in the actually resected left SMA (Figure 9c). The nonlinear excess interactions prior to seizure 2 were widely distributed without any star‐like, centric structure (Figure 9d), which might further explain the unfavorable outcome and does not allow to hypothesize that outcome might have been better if the left mesiotemporal structures were also removed in this patient.

In all cases, our analysis suggests the resection of additional brain areas. If resection beyond the clinical gold standard is feasible is highly dependent on the specific case and must be assessed by physicians and patients under consideration of the expected benefits and impairments. In some cases, this kind of additional information could lead to larger or different, more promising resections whereas in others it could help to preclude unhelpful surgery. Both would be highly beneficial for patients if available early on.

### 4.3 Relation to other work

In general, network results based on signal interrelations are always specific to the applied dependence measure and rather complement than invalidate each other. It has repeatedly been stated that linear and nonlinear measures for signal interrelation yield qualitatively similar results when applied to simulated signals or real iEEG (Ansari‐Asl, Senhadji, Bellanger, & Wendling, 2006; Kreuz et al., 2007; Mormann et al., 2005; Osterhage, Mormann, Wagner, & Lehnertz, 2007; Wendling, Ansari‐Asl, Bartolomei, & Senhadji, 2009). One reason might be that, in contrast to our correction using multivariate IAAFT surrogates, these did not explicitly account for the fact that nonlinear measures are also sensitive to linear dependences, which are often the dominant ones. In contrast but consistent with earlier findings, we observed a fundamental difference between surrogate‐corrected linear and nonlinear interrelation networks. Andrzejak and collaborators used a different surrogate‐corrected nonlinear interrelation measure to analyze continuous interictal data. They congruently found stronger nonlinear excess interrelations in the focal hemisphere (Andrzejak et al., 2011). In a subsequent study, they found more rejections of a nonlinear‐independence test for interictal EEG recorded from the SOZ than for nonfocal channels (Andrzejak et al., 2012). Signals from epileptogenic brain areas also were more stationary and less random. They hypothesized that epilepsy strengthens the nonlinear portion in the superposition of linear and nonlinear dynamics present in the EEG signals.

Regarding network analysis of iEEG a large amount of studies have been undertaken in the past decade (see, e.g., Lehnertz, Geier, Rings, & Stahn, 2017; Parvizi & Kastner, 2018 for reviews). Directed graphs have been analyzed, for instance, by Wilke et al. (2011), van Mierlo et al. (2013), and Zubler et al. (2015). Here, we focused on undirected interrelations. Multiple studies also analyzed linear cross‐correlation networks derived from iEEG (Khambhati et al., 2015; Shah et al., 2019; Warren et al., 2010). They all found linear connections inside the SOZ to be stronger than those outside the SOZ or bridging these areas. Moreover, Warren and colleagues also found linear connections outside the SOZ to be stronger than the bridging connections (Warren et al., 2010).

Ortega, de la Prida, Sola, and Pastor (2008) studied intraoperative electrocorticography recordings of temporal lobe epilepsy patients. They calculated Pearson's correlation coefficient and mutual information and tested interrelations for significance using independent shift surrogates for all channels. They observed that removal of sharply defined synchronization clusters was correlated with seizure control. In contrast to our study, they did not find a systematic difference between the results from the linear and nonlinear interrelation measures. Our analyses using shift surrogates (see Figure S3) showed very high similarity to the linear matrices and suggest that the most likely explanation for this is that they did not use multivariate surrogates to correct for linear interrelation to which mutual information is also sensitive. The methodology introduced by Rummel et al. (2011) and Andrzejak et al. (2011) is capable of explicitly quantifying the nonlinear excess interrelation, reaching beyond linear effects.

Several EEG‐based studies found indications for a functional decoupling of the focal zone around seizure onset (Kramer et al., 2010; Rummel et al., 2013; Schindler et al., 2010; Schindler, Gast, Goodfellow, & Rummel, 2012; Warren et al., 2010; Wendling, Bartolomei, Bellanger, Bourien, & Chauvel, 2003). This is in line with the results from our linear interrelation analyses: The hemispheres are decoupled from each other (weak linear connections between hemispheres) and more specifically the resected brain tissue is decoupled from the remainder (weak linear bridging connections). Beyond that, this view is enriched here by the nonlinear excess interrelations showing a different picture: Centered in the resected brain tissue and in the focal hemisphere, they connect to virtually all other brain areas we were recording from, also between both hemispheres. The clearly increased occurrence of nonlinear excess interrelations in the focal channels is again in agreement with former findings (Andrzejak et al., 2001, 2006, 2011, 2012; Casdagli et al., 1997) and supports the hypothesis that excessive nonlinear activity is a hallmark of epileptogenic activity rather than simply a characteristic of the mesial structures of the temporal lobe, namely the hippocampi and the amygdalae.

## 5 CONCLUSION AND OUTLOOK

In this study, we found fundamentally different properties of linear and nonlinear iEEG interrelation networks when corrected by appropriate surrogates. Linear interrelation patterns are organized locally in block‐like manner and highly reproducible between patients. In contrast, nonlinear excess networks have star‐like structure with interactions overrepresented in the focal brain regions and are specific to the individual pathology. This makes them a candidate mechanism to efficiently transmit information from the focal zone to distant brain areas or alternatively to act as compensatory mechanism toward the focal zone, an important research question that needs further investigation. Another subject to address in future studies is about the mechanisms underlying these long‐range nonlinear excess interactions. Two potential candidates might be neuronal burst firing (Cain & Snutch, 2013) and nonsynaptic electric field coupling (Shivacharan, Chiang, Zhang, Gonzalez‐Reyes, & Durand, 2019).

Regarding epilepsy surgery, our results corroborate the potential of network analysis to support identification of the epileptogenic zone using either linear interrelations to measure its decoupling or the excessive presence of nonlinear excess interrelations. Combination of these two approaches to enhance the overall accuracy is planned to be investigated in upcoming projects.

## CONFLICT OF INTEREST

None of the authors has any conflict of interest to disclose.

## Supporting information


**Appendix S1:** Supporting information

## Data Availability

We cannot share the data because this would compromise the confidentiality confirmation. In a small single center study, it would jeopardize the anonymity of the patients. The code to create the linear and nonlinear excess matrices is available on GitHub (https://github.com/chrummel/XMImatr_v2_plosone). Additional code may be shared upon direct request. The code sharing complies with the requirements of the institute and with institutional ethics approval.
