# Supplementary material for: Linear and nonlinear interrelations show fundamentally distinct network structure in preictal intracranial EEG of epilepsy patients
Source: Hum Brain Mapp. 2019 Oct 18;41(2):467–83. doi: 10.1002/hbm.24816 (PMC7268049; doi:10.1002/hbm.24816)
Supplement: Supplementary file 1 — Appendix S1: Supporting information [file HBM-41-467-s001.pdf]

Linear and nonlinear interrelations show fundamentally distinct network structure in preictal intracranial EEG of epilepsy patients

Supplementary Material

Michael Müller[1,2], Matteo Caporro[2], Heidemarie Gast[2], Claudio Pollo[3], Roland Wiest[1], Kaspar Schindler[2], and Christian Rummel *[1]

[1]Support Center for Advanced Neuroimaging (SCAN), University Institute for Diagnostic and Interventional Neuroradiology, Inselspital, Bern, Switzerland
[2]Department of Neurology, Inselspital, Bern University Hospital, University Bern, Bern, Switzerland
[3]Department of Neurosurgery, Inselspital, Bern University Hospital, University Bern, Bern, Switzerland

*Corresponding author: Support Center for Advanced Neuroimaging (SCAN), University Institute for Diagnostic and Interventional Neuroradiology, Inselspital, Freiburgstrasse 4, 3010 Bern, Switzerland, Tel.: +41 (0)31 632 80 38, Email: crummel@web.de

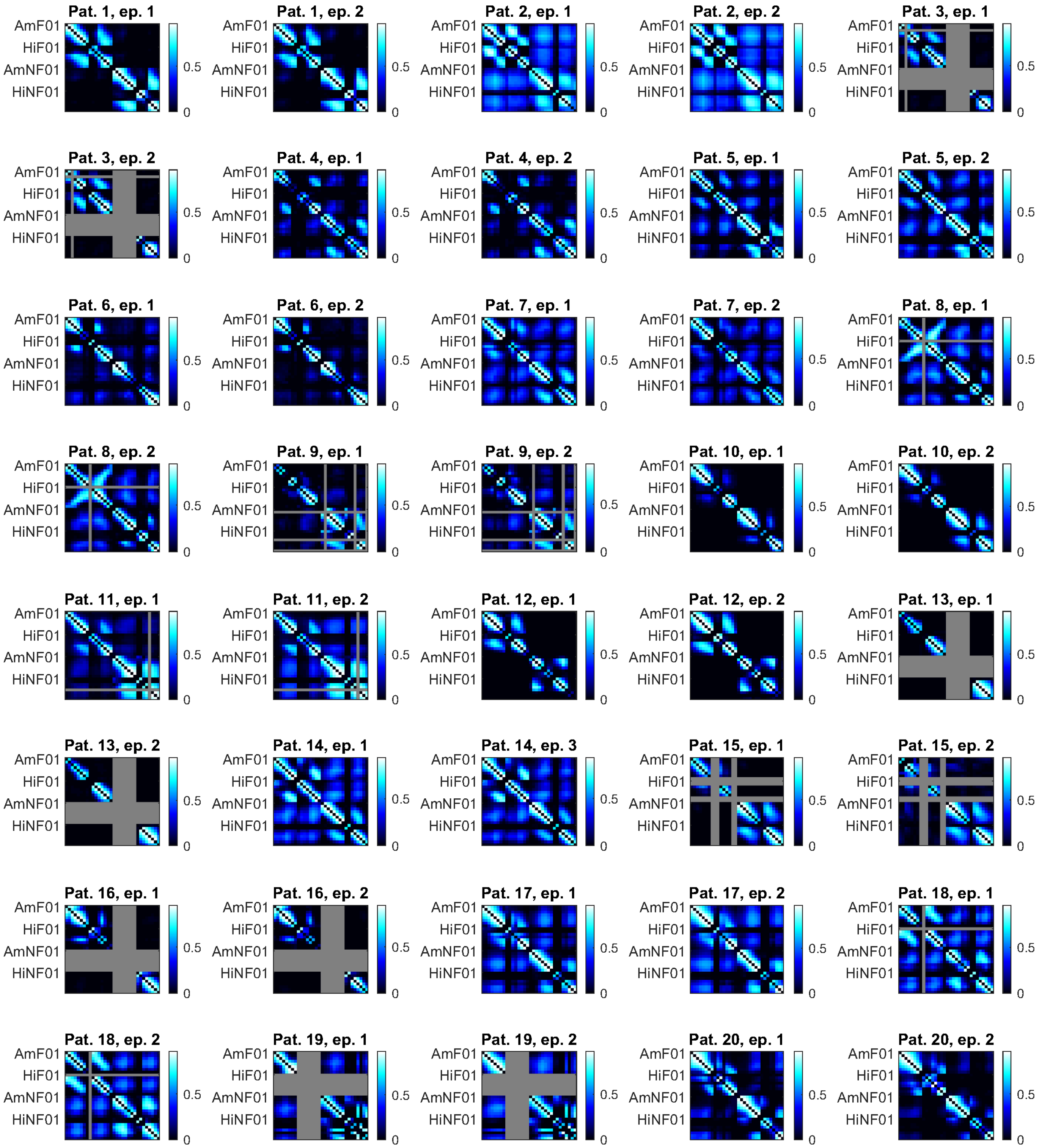

Figure S1: Epoch-wise averages of all univariate IAAFT surrogate corrected linear interrelation matrices of both preictal analysed epochs of all patients. All channels being part of the standardized implantation scheme are shown, channels of this scheme not present in a patient are displayed in grey to be clearly distinguished from colors appearing in both chosen color scales. The order of the electrodes is laterality-matched. A high intra- and inter-patient reproducibility exists. Abbreviations: Pat.: patient, ep.: epoch, Am*: electrode recording from the amygdala, Hi*: electrode recording from the hippocampus, F: focal, NF: non-focal.

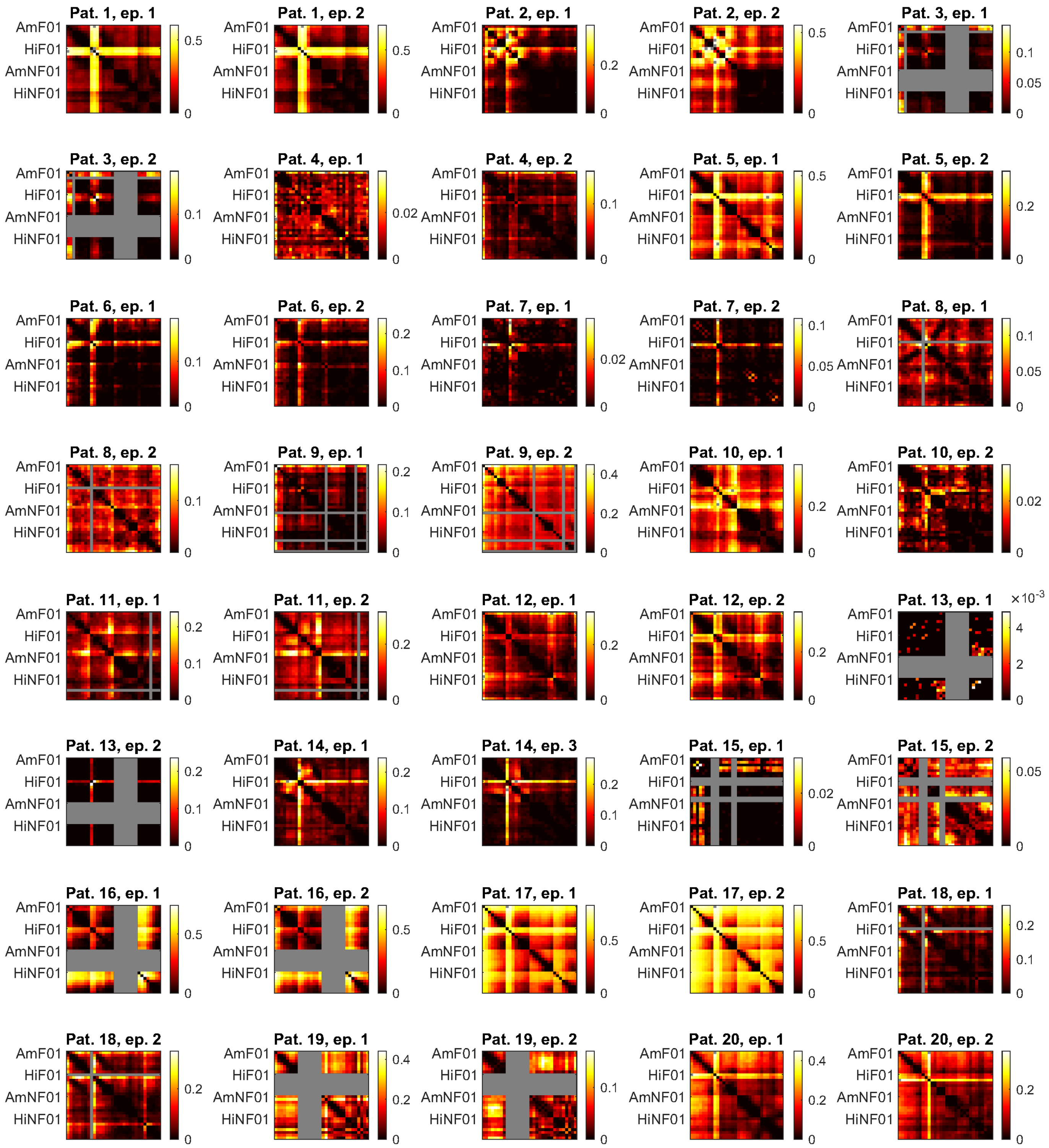

Figure S2: The same as in Figure S1 for the multivariate IAAFT surrogate corrected nonlinear interrelation matrices. Multivariate IAAFT surrogates preserve the linear cross-correlation between signals but not their nonlinear autocorrelation. In this case, only high intra-patient reproducibility exists.

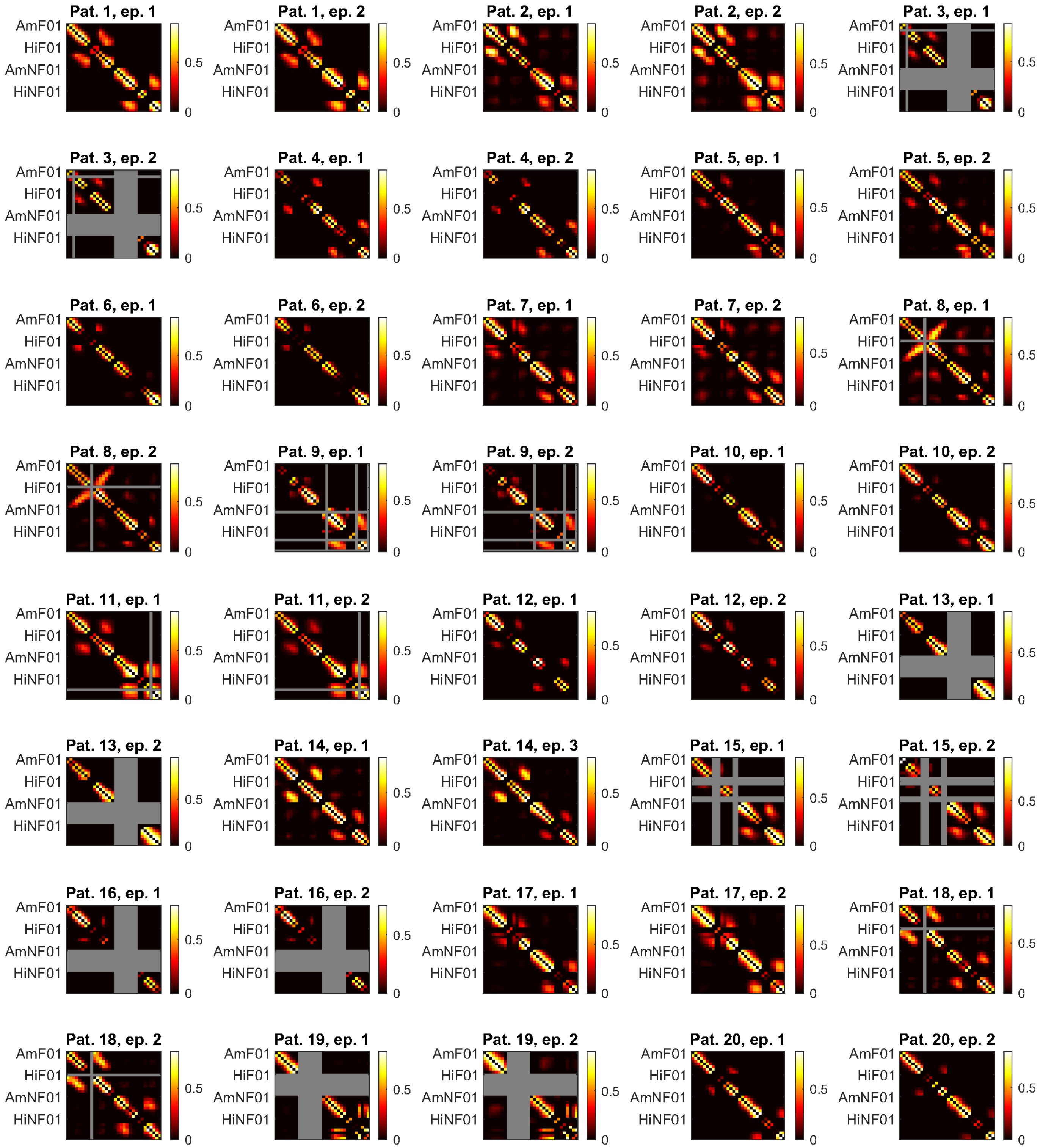

Figure S3: The same as in Figure S2 for the shift surrogate corrected nonlinear interrelation matrices. Shift surrogates preserve the nonlinear autocorrelation of the signals but not the dependences between them. The resulting matrices look very similar to the linear ones with a high intra- and inter-patient reproducibility. **This can be interpreted as an indication that the linear cross-correlation is the dominant factor in the nonlinear interrelations. Further tests are necessary to prove this conjecture.**

		Linear			Nonlinear		
Patient	epoch	# in core	in F	in R	# in core	in F	in R
1	1	25	0.52	0.16	3	1.00	1.00
1	2	27	0.51	0.19	3	1.00	1.00
2	1	30	0.53	0.17	1	1.00	1.00
2	2	31	0.52	0.16	1	1.00	1.00
3	1	22	0.64	0.36	2	1.00	1.00
3	2	22	0.64	0.36	5	1.00	1.00
4	1	17	0.47	0.24	1	1.00	1.00
4	2	10	0.30	0.10	1	1.00	1.00
5	1	2	0	0	2	1.00	1.00
5	2	2	0	0	2	1.00	1.00
6	1	25	0.48	0.28	1	1.00	1.00
6	2	25	0.48	0.28	2	1.00	1.00
7	1	30	0.50	0.10	1	1.00	1.00
7	2	30	0.50	0.10	1	1.00	1.00
8	1	15	0.60	0.20	1	1.00	1.00
8	2	21	0.57	0.24	3	0.67	0.33
9	1	9	0.22	0	1	1.00	1.00
9	2	8	0.25	0	1	1.00	1.00
10	1	29	0.52		4	1.00	
10	2	6	0		1	1.00	
11	1	3	0		4	1.00	
11	2	23	0.43		2	1.00	
12	1	17	0.59		1	1.00	
12	2	17	0.65		2	1.00	
13	1	22	0.68	0.09	7	0.43	0
13	2	22	0.68	0.09	1	1.00	0
14	1	30	0.53	0.47	1	1.00	1.00
14	2	29	0.55	0.48	1	1.00	1.00
15	1	26	0.42		3	1.00	
15	2	26	0.42		3	1.00	
16	1	22	0.64		3	0	
16	2	22	0.64		1	0	
17	1	27	0.52		4	1.00	
17	2	27	0.52		4	1.00	
18	1	27	0.48		2	1.00	
18	2	30	0.46		2	1.00	
19	1	17	0.41		1	0	
19	2	19	0.42		16	0.44	
20	1	1	1.00		1	1.00	
20	2	31	0.52		1	1.00	

Table SI: Of all epoch-wise averaged IAAFT surrogate corrected linear and nonlinear interrelation matrices, the number of automatically identified core nodes of the standardized implantation scheme (# in core), their fraction in the focal hemisphere (in F), and for patients who got surgery in the mesiotemporal areas their fraction in the resected brain tissue (in R).

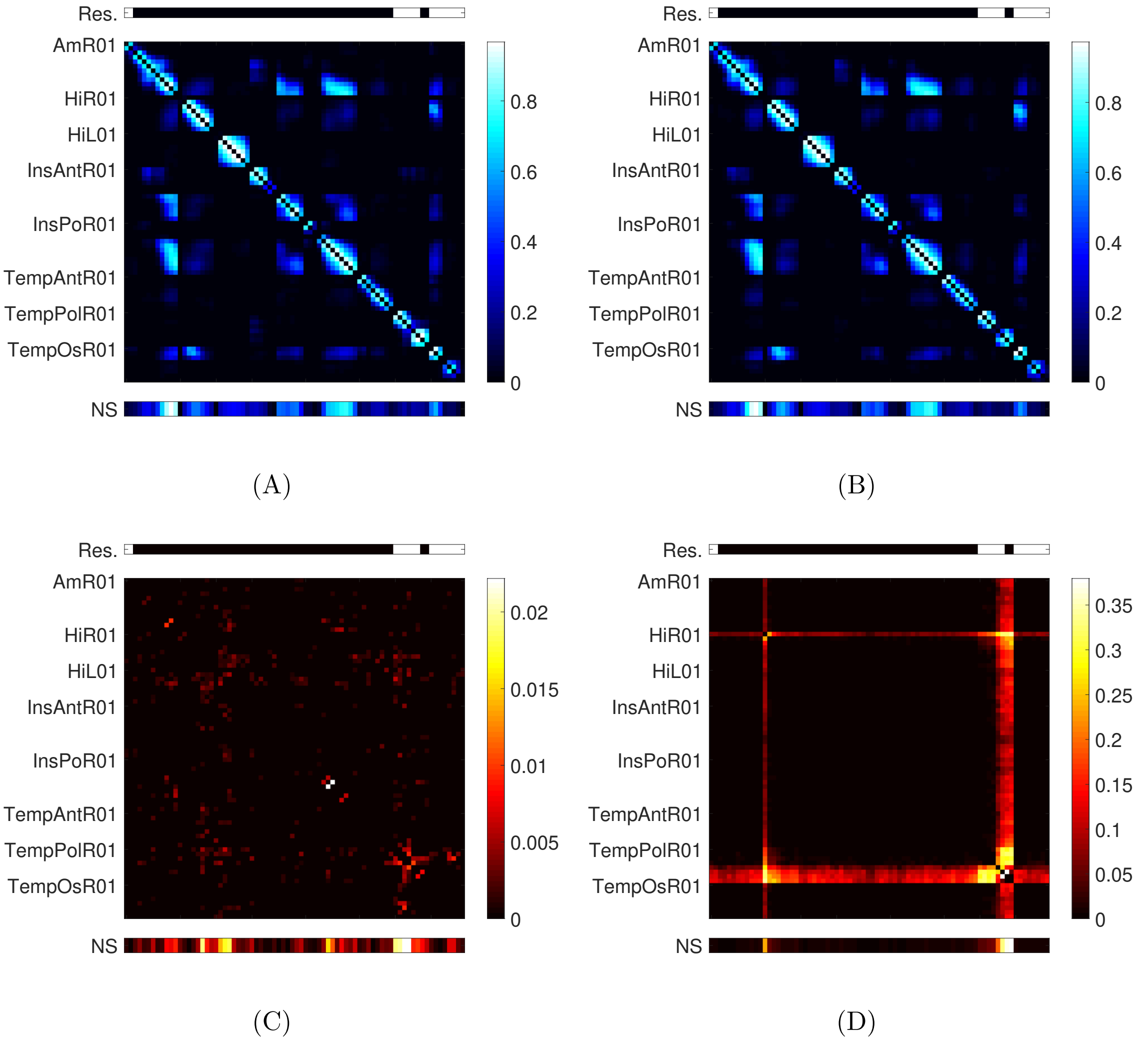

Figure S4: Preictal averages of the IAAFT surrogate corrected linear (A,B) and nonlinear (C,D) interrelation matrices before seizure 1 (A,C) and seizure 2 (B,D) of patient 13. All channels of all electrodes implanted in this patient are shown. Above the matrices the channels recording from brain tissue that was later resected are indicated by white bars. Below the matrices the node strengths are displayed.

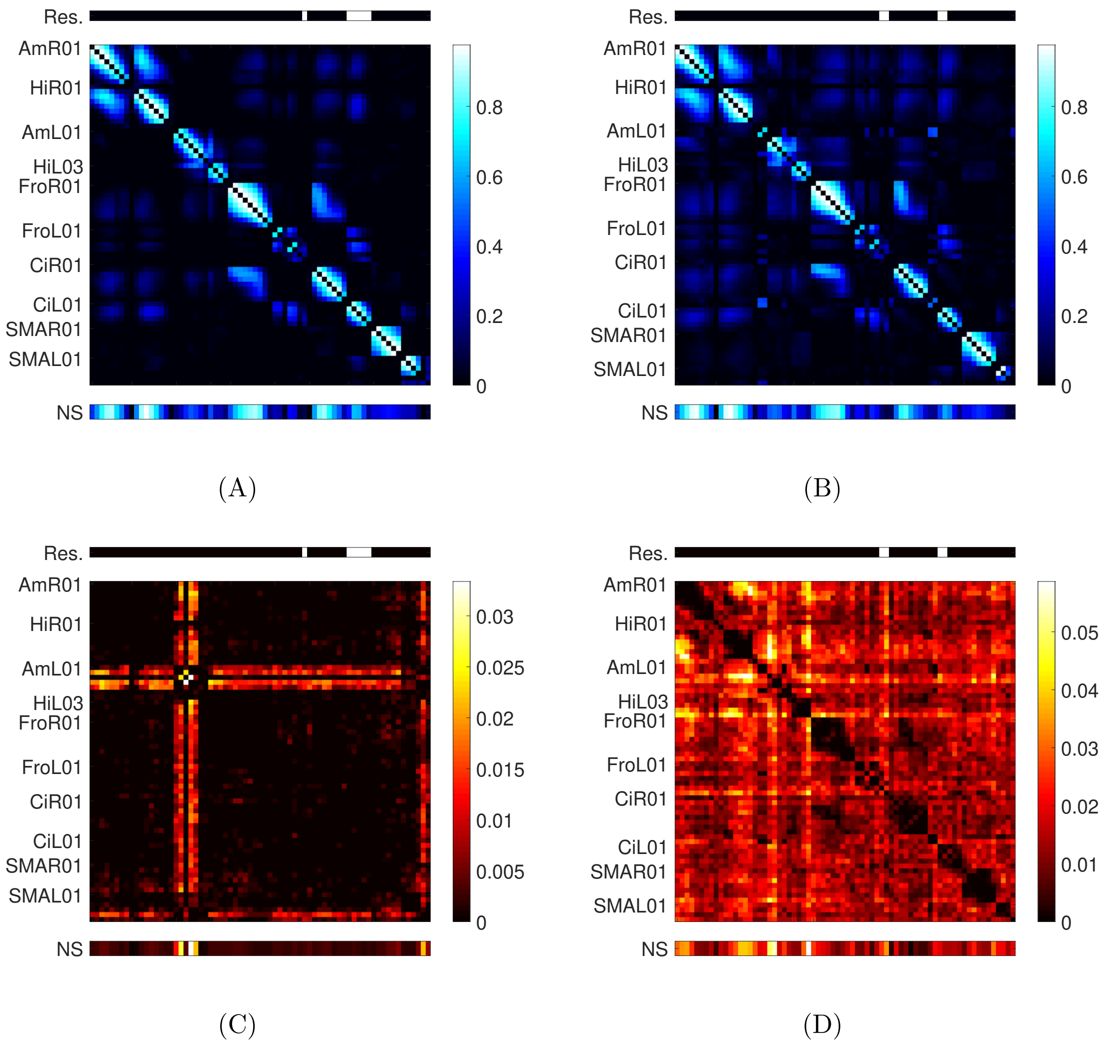

Figure S5: Preictal averages of the IAAFT surrogate corrected linear (A,B) and nonlinear (C,D) interrelation matrices before seizure 1 (A,C) and seizure 2 (B,D) of patient 15. In both seizures, few (but different) channels had to be excluded due to artifacts. Above the matrices the channels recording from brain tissue that was later resected are indicated by white bars. Below the matrices the node strengths are displayed.
